# Electrical impedance myography for the detection of muscle inflammation induced by λ-carrageenan

**DOI:** 10.1371/journal.pone.0223265

**Published:** 2019-10-01

**Authors:** Marie Mortreux, Carson Semple, Daniela Riveros, Janice A. Nagy, Seward B. Rutkove

**Affiliations:** Department of Neurology, Beth Israel Deaconess Medical Center, Harvard Medical School, Boston, Massachusetts, United States of America; University of Florida, UNITED STATES

## Abstract

Electrical impedance myography (EIM) is a technique for the assessment of muscle health and composition and has been shown to be sensitive to a variety of muscle pathologies including neurogenic atrophy and connective tissue deposition. However, it has been minimally studied in pure inflammation. In this study, we sought to assess EIM sensitivity to experimental inflammation induced by the localized intramuscular injection of λ-carrageenan. A total of 91 mice underwent 1–1000 kHz EIM measurements of gastrocnemius using a needle array, followed by injection of either 0.3% λ-carrageenan in phosphate-buffered saline (PBS) or PBS alone. Animals were then remeasured with EIM at 4, 24, 48, or 72 hours and euthanized and quantitative assessment of muscle histology was performed. Parallel alterations in both 5 and 50 kHz EIM values were identified at 4 and 24 hours, including reductions in phase, reactance, and resistance. In PBS-treated animals these values normalized by 48 hours, whereas substantial reductions in phase and reactance in 5 kHz EIM values persisted at 48 and 72 hours (i.e., values of phase 72 hours post-injection were 6.51 ± 0.40 degrees for λ-carrageenan versus 8.44 ± 0.35 degrees for PBS p<0.001, n = 11 per group). The degree of basophilic area observed in muscle sections by histology correlated to the degree of phase change at these two time points (R_spearman_ = -0.51, p = 0.0029). Changes in low frequency EIM parameters are sensitive to the presence of inflammatory infiltrates, and have the potential of serving as a simple means of quantifying the presence and extent of muscle inflammation without the need for biopsy.

## Introduction

A variety of diagnostic tools exist to evaluate muscle inflammation, whether due to primary disease or injury. These include imaging techniques, such as magnetic resonance imaging (MRI) [1], ultrasound [1,2], and more recently, positron emission tomography (PET scanning) [3]. Of course, primary histological analysis can also be performed, with certain stains geared specifically for evaluating various types of inflammatory cells, including macrophages and lymphocytes [4]. Each of these approaches has its own specific advantages and disadvantages. One approach that holds potential promise but has not been studied specifically for this purpose is electrical bioimpedance. In most bioimpedance methods, a low-amplitude, high frequency electrical current is passed between two electrodes overlying a body region of interest and consequent surface voltages measured [5]. Alterations in the health and composition of the tissue will alter the relationship between the resultant voltages, thus providing information on the state of the tissue. The specific application of impedance methods to muscle, termed electrical impedance myography (EIM), has certain potential advantages in that it can be applied non-invasively at the bedside and provides quantitative data on tissue health without the need for complex image analysis [6]. The technique has been studied in a variety of disorders that affect muscle, ranging from muscular dystrophy [7] to amyotrophic lateral sclerosis [8]. With the exception of one small clinical data set [9], the technique has not been studied carefully in muscle disorders characterized by inflammation. The technique’s specific value in this context is that of serving as a convenient biomarker for future clinical trials in disorders in which muscle inflammation plays a major role, such as autoimmune myositis.

In this study, we sought to evaluate the potential of EIM in inflammatory conditions by performing a carefully modeled study in mice using a standard pro-inflammatory molecule, λ-carrageenan, a linear sulfated polysaccharide extracted from red edible seaweed [10,11], and assessing the time course of impedance changes and the relationship between EIM changes and muscle histology. For theoretical reasons, as discussed below, we anticipated that the change in impedance characteristics would be most evident at lower frequencies (in the 1–10 kHz range) given the extracellular nature of this particular pathological process. Thus, in this study we chose a low frequency value of 5 kHz, and for comparison, the more standard frequency of 50 kHz, which has been included in most of our EIM research completed to date.

## Materials and methods

### Animals

All experimental protocols were approved by the Beth Israel Deaconess Medical Center Institutional Animal Care and Use Committee. Ninety-one C57Bl/6J 10-week old male mice weighing 27.23 ± 0.18 g were obtained (Jackson Laboratory) and housed in a temperature-controlled animal facility (22 ± 2°C) room with a 12:12-h light-dark cycle. Water and regular chow were provided *ad libitum*.

### PBS and λ-carrageenan-induced inflammation studies

Mice were anesthetized using a vaporizer (1.5–3.5% isoflurane + oxygen), and body-temperature was maintained constant using a water-blanket (Gaymar, Inc., Orchard Park, NY, USA) set at 37°C (Fisher Scientific, Hampton, NH, USA) for the entire duration of the procedure. Animals were positioned in a prone position, and the left leg was taped securely at a 45° angle. The limb was shaved using a hair clipper (Braintree Scientific, Braintree, MA, USA). Baseline EIM parameters of the gastrocnemius-soleus complex (triceps surae) were obtained. Animals were then injected intramuscularly with 10 μL of phosphate buffered saline (PBS) (Research Product International, Mount Prospect, IL, USA) or 10 μL of 0.3% λ-carrageenan (Sigma-Aldrich, St-Louis, MO, USA) in PBS across a total of 2–4 injection sites longitudinally using a 10 μL Hamilton syringe (701SN, Hamilton Company, Reno, NV, USA). Animals were returned to their cages and assessed with EIM at 4, 24, 48, or 72 hours and then euthanized for histological examination (each animal undergoing only a pre-injection and single post-injection assessment). The animals’ wellbeing was monitored regularly to ensure that none were exhibiting signs of distress.

### Electrical Impedance Myography (EIM)

*In vivo* impedance measurements of the left gastrocnemius were performed under 1.5% isoflurane anesthesia using the mView System (Myolex, Inc, Boston, MA, USA), and obtained at 41 frequencies ranging from 1 kHz to 10 MHz. However, only frequencies between 2 kHz and 1 MHz were included in our planned analysis given anticipated artifacts that occur at the extremes of the frequency spectrum, yielding a total of 29 frequencies for analysis. Measurements were made using a fixed 4 mm wide needle array (2 mm deep, 1 mm of the distal tips left exposed) that was inserted along the length of the left gastrocnemius muscle. The array was designed from a series of 27G subdermal needle electrodes (Ambu, Neuroline, Copenhagen, Denmark). We coated the needles evenly with standard nail polish leaving only the tips exposed to help ensure a restricted region of current flow and voltage measurement.

### Harvest and tissue processing

Animals were euthanized following IACUC guidelines. The entire left gastrocnemius-soleus complex was excised and weighed on an analytical scale (Fisher Scientific, Pittsburgh, PA, USA). The muscle complex was then fixed in 10% formalin for 48 hours at 4°C and transferred to PBS and embedded in paraffin. Paraffin sections were stained using regular H&E procedure and the basophilic area was quantified using a color deconvolution process, with experimenters blinded to treatment and time (FIJI, ImageJ, NIH).

### Statistical analyses

Data were analyzed using Graphpad Prism 8 (Graphpad Software, La Jolla, CA), using the most appropriate tests (unpaired t-tests or one-way ANOVA followed by post-hoc tests). Correlation analyses were performed using Spearman’s r. Results are displayed as mean ± SEM unless stated otherwise, and were considered significant at p<0.05.

## Results

### Effects of λ-carrageenan vs PBS on EIM parameters at 5 and 50 kHz

**Fig 1:**
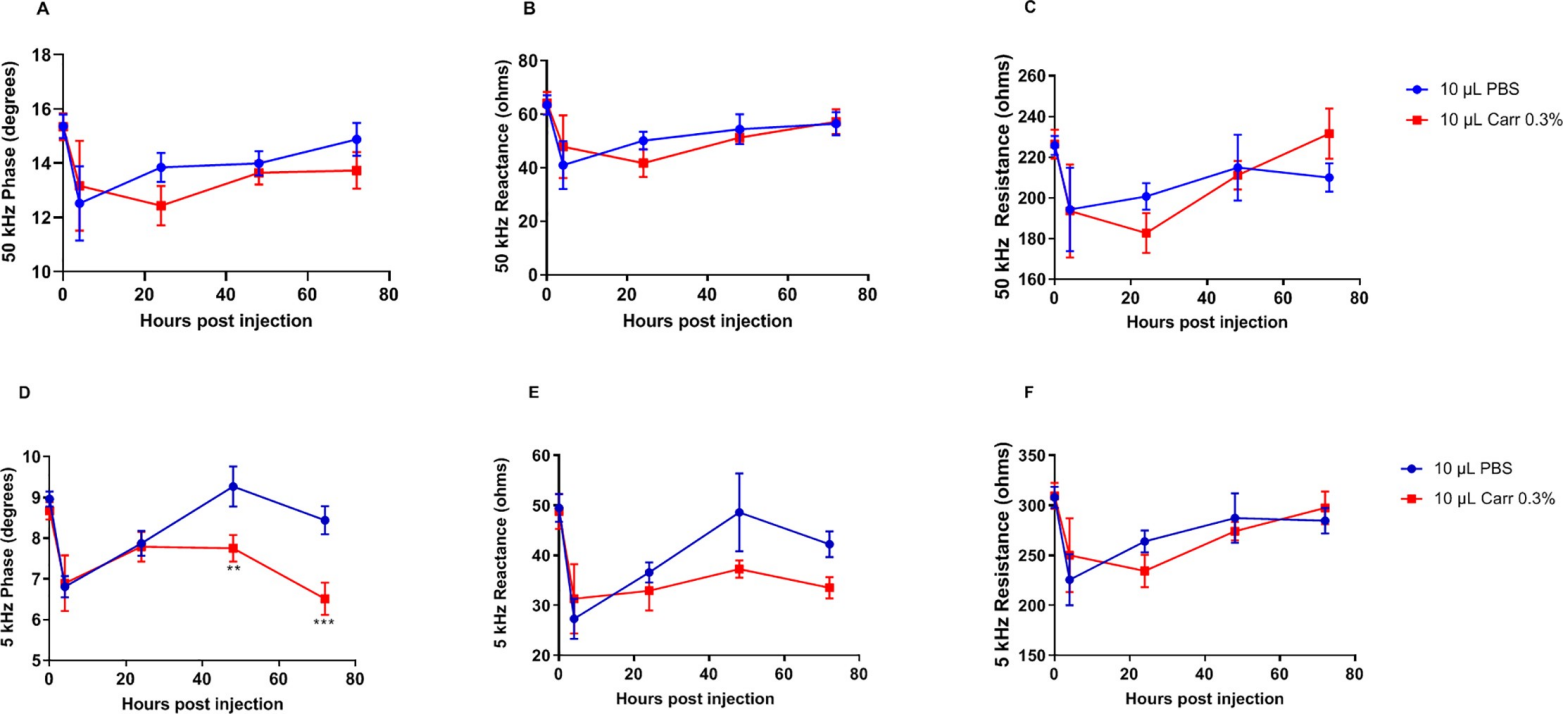
Evolution of longitudinal EIM parameters. Evolution of longitudinal EIM parameters at 50 kHz (A-C) and 5 kHz (D-F), following injection of PBS or of 0.3% λ-carrageenan (0.3% carr), including phase (A, D), reactance (B, E), and resistance (C, F). n = 5–13 per group. **: p<0.01 and ***: p<0.001 vs PBS group at the same time-point.

Fig 1 and Table 1 show a comparison of the EIM parameters (i.e., phase, reactance and resistance) obtained following PBS versus λ-carrageenan injection at both 5 and 50 kHz. Given the traumatic nature of injecting PBS alone into the muscle, as well as the anticipated impedance alterations due to the presence of PBS, it was critical that we assessed the impact of these effects as a control. At 50 kHz, EIM values for both λ-carrageenan and PBS follow similar trajectories, showing a clear change within the first 24 hours that begins to normalize at 48 and 72 hours (Fig 1A, 1B and 1C). In contrast, for reactance and phase, at 5 kHz, this parallel behavior continues only out to 24 hours, at which point PBS treatment mostly returns to baseline whereas λ-carrageenan treatment shows increasing departure from normal (Fig 1D and 1E). Resistance following λ-carrageenan treatment, in contrast, mostly follows in parallel to PBS (Fig 1F).

**Table 1:** Temporal evolution of the EIM parameters at 5 kHz.

	PBS-treated			λ-carrageenan treated		
	Phase (±SEM)	Reactance (±SEM)	Resistance (±SEM)	Phase (±SEM)	Reactance (±SEM)	Resistance (±SEM)
**Pre-injection**	8.97±0.18	49.50±2.78	308.12±10.49	8.68±0.21	48.77±3.47	309.55±13.07
**4 hours**	6.81±0.26	27.35±4.01	225.73±25.58	6.90±0.69	31.34±6.95	250.24±36.80
**24 hours**	7.88±0.30	36.60±2.01	264.14±10.86	7.79±0.36	32.95±3.98	234.58±16.17
**48 hours**	9.27±0.49	48.62±7.78	287.50±24.74	7.76±0.33**	37.28±1.74	274.31±3.17
**72 hours**	8.44±0.35	42.26±2.60	284.73±12.75	6.51±0.40***	33.55±2.14	297.62±16.38

**: p <0.01

***: p<0.001 vs PBS-treated.

### Multifrequency data

**Fig 2:**
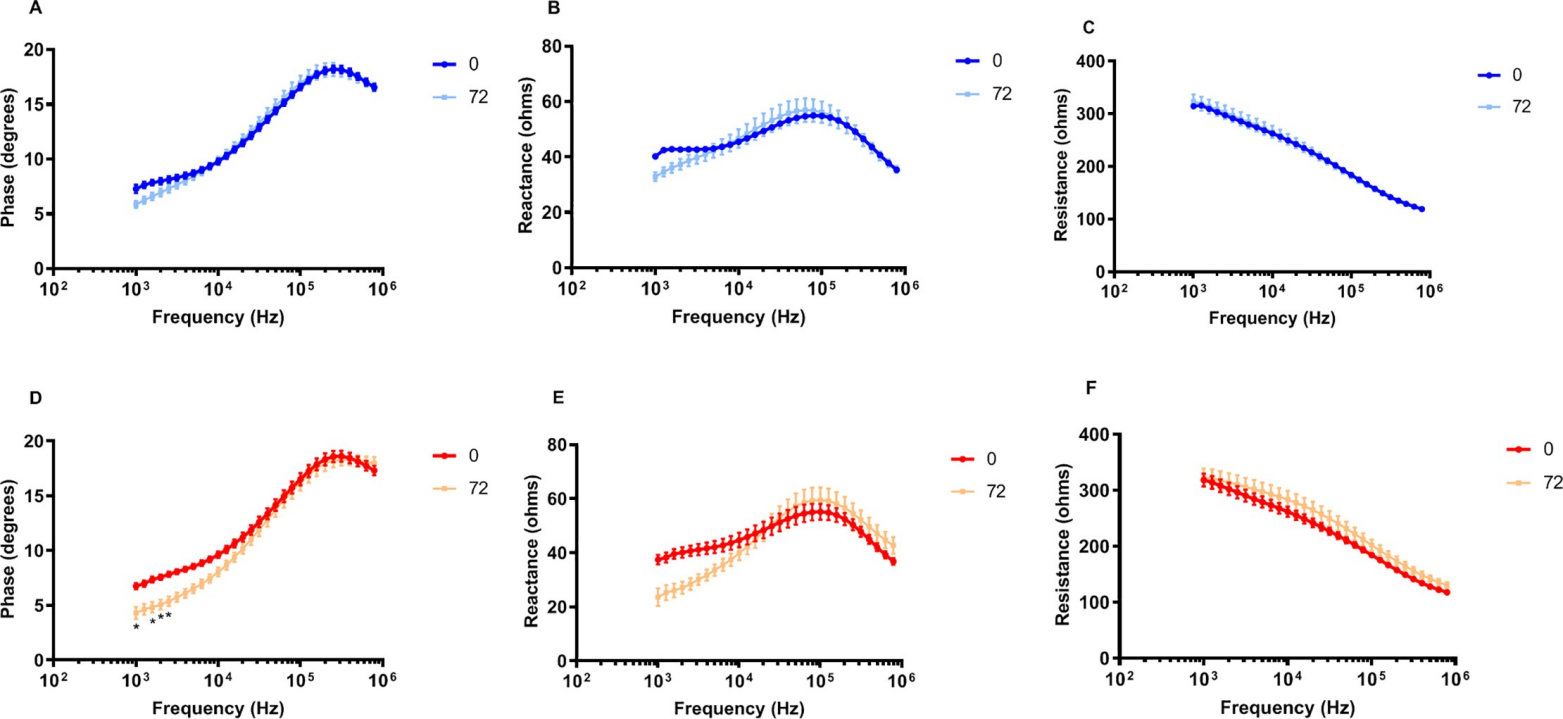
Multifrequency analysis of the EIM parameters. Phase (A, D), reactance (B, E), and resistance (C, F) in PBS-injected mice (A-C) and λ-carrageenan-injected mice (D-F), pre-injection and 72 hours post injection, n = 11 per group. Data were analyzed using repeated measures 2-way ANOVA and the results of the post hoc tests are represented as *: p<0.05.

Fig 2 shows the overall impedance spectra for phase, reactance, and resistance (between 1 and 1000 kHz) at 0 and 72 hours averaged for the entire group of animals (PBS in blue (A-C) and λ-carrageenan in red (D-F). As can be seen for all EIM parameters, the overall changes are modest between the two time points, with the low-frequency region (<10^4^ Hz) showing the greatest change. Interestingly, the PBS animals (2A, B) show some change in both phase and reactance in that frequency region as well, possibly reflecting some injury/trauma related to the needle insertion (but which would not have been captured in our basophilic histological analysis below).

### Relationship between EIM and histological analyses

**Fig 3:**
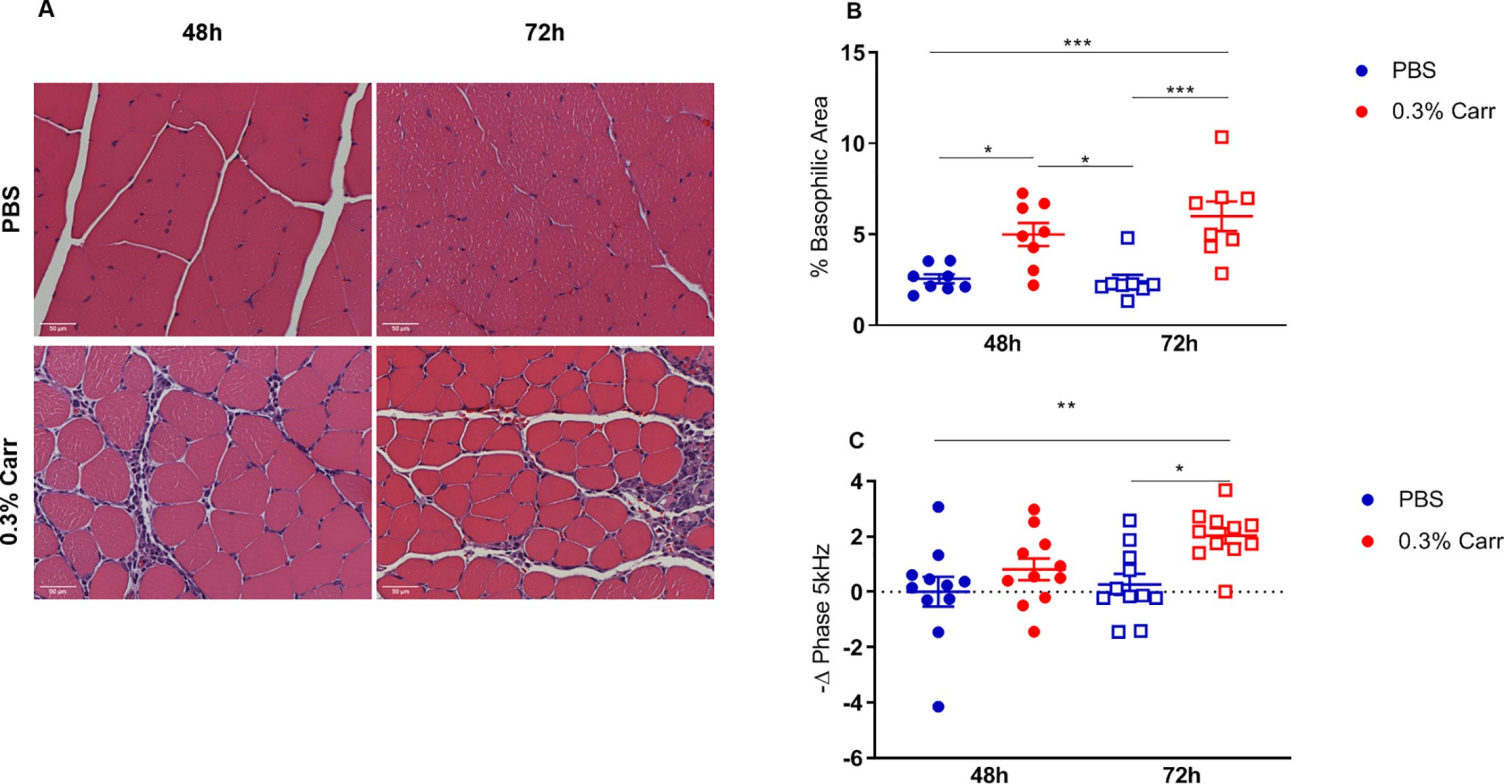
Histological quantification of inflammation. (A) Representative images (40x) of the gastrocnemius muscle stained with H&E at 48h and 72h after PBS or 0.3% λ-carrageenan injection. (B) Quantification of the basophilic stained cells in the gastrocnemius muscle. (C) Change in phase at 5 kHz 48 and 72 hours post injection compared to pre-injection values. The results of Tukey’s post hoc test are represented as *, **, and ***: p<0.05, p<0.01 and p<0.001, respectively, n = 8 per group. Bar equals 50 microns.

Fig 3A shows prototypical histological data from two mice at 48 and 72 hours after injection with PBS (frames A, B) and after treatment with λ-carrageenan (frames C, D). As can be observed in Fig 3A, there is a considerable inflammatory infiltrate (as evidenced by an increase in basophilic staining area) developing at these later time points in the animals receiving λ-carrageenan. Fig 3B shows quantified % basophilic stained area comparing PBS versus λ-carrageenan and Fig 3C shows the relative change in phase at 5 kHz (comparing baseline to 48- and 72-hour time points). As can be observed, the changes between phase and basophilic area parallel each other.

**Fig 4:**
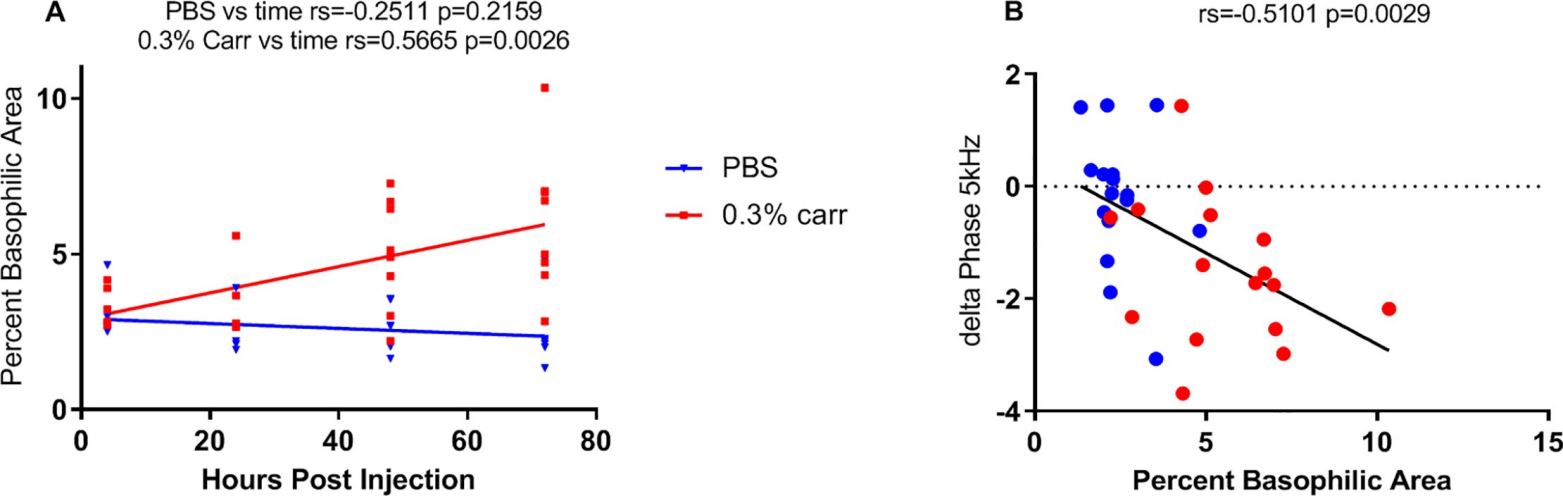
Correlations of EIM parameters and histological analysis with treatment. (A) Correlations between treatment and basophilic area at all time-points (n = 5–8 per group). (B) Correlation between the change in phase at 5 kHz and basophilic area at 48 and 72 hours post injection (n = 16 per group). Correlations were analyzed using Spearman’s test.

This concept is further explored in Fig 4A in which the % basophilic area is plotted as a function of time for the PBS- and λ-carrageenan-treated animals (at 4, 24, 48, and 72 hours). As can be seen in Fig 4A, there is no increase in % basophilic area over this time period in the mice receiving PBS, indicating no change in the number of myofiber nuclei and the absence of inflammatory infiltrates. However, there is a clear increase (i.e., 78.2%) in the basophilic area from 4 to 72 hours in animals receiving λ-carrageenan, representing the influx of an inflammatory response. Furthermore, we observed a significant correlation between the basophilic area and time following injection in the λ-carrageenan-treated mice (Rs = 0.566, p = 0.0026), and no significant correlation in the PBS-treated animals (Rs = -0.251, p = 0.2159, n = 5–8 per group). Finally, Fig 4B, shows a correlation between % basophilic area at 48 and 72 hours (the times in which the most inflammatory cell infiltration has developed) and the change in phase at 5 kHz from baseline for each individual animal. As anticipated, there was a significant correlation between the two values, with R = -0.51, p = 0.0029).

## Discussion

Injection of 10 μL of 0.3% λ-carrageenan into the gastrocnemius-soleus complex successfully induced an inflammatory response, which was detected using low frequency EIM parameters as we had predicted based on impedance theory. Among them, the phase seems to be the most sensitive EIM parameter to inflammation and displays a continuous decline up to 72 hours post-injection of λ-carrageenan. The progressive decrease in phase values is associated with an increased number of basophilic cells into the muscle tissue, largely due to neutrophil infiltration.

Prior to undertaking this study, we had tested different injection volumes and different λ-carrageenan concentrations. We observed that an injection of only 5 μL of 0.3% λ-carrageenan (one-half the volume reported here) yielded a very small response in the EIM parameters. Alternatively, an injection of 20 μL of 0.3% λ-carrageenan (twice the volume reported here) resulted in considerable variability in the data, possibly related to the fact that this volume was greater than the capacity of the tissue and reflux from the injection site was often observed. Previous studies have used λ-carrageenan at several different concentrations[12–14].Thus, in addition to the 0.3% concentration used here, we also studied a 3% solution, but its high viscosity prevented its being easily injected using a 29G syringe.

Our specific interest in low frequency changes (i.e., 5 kHz) in EIM parameters and prediction that this is the frequency range in which the greatest changes induced by inflammation would be observed, follows from standard electrical bioimpedance theory. At low frequencies, the electrical current cannot readily pass through cell membranes (the main contributor to the measured reactance), thus intracellular effects are not typically observed. More critically, the inflammatory infiltrates themselves can be viewed as single large muscle inclusions with impedance characteristics that are distinct from the surrounding myofibers. These inclusions are likely associated with edema (not identifiable on pathological analysis) and thus it is likely that these microscopic regions of extracellular change are being detected by our needle array. While the current will still flow through such regions at the higher “standard” EIM frequency of 50 kHz, it is flowing through so much more of the tissue, that the change is no longer detectable.

Unlike much of our typical earlier animal work [15], we specifically used a needle electrode array rather than a surface electrode array in the present work. The main reason for doing so was related to the short follow-up needed after the initial injection of either PBS alone or PBS containing λ-carrageenan. For surface measurements, it is necessary to thoroughly remove the fur using a depilatory agent [16]. While effective for immediate impedance measurements, over a period of hours the skin subsequently develops edema that produces reductions in the impedance data. These alterations can last for several days and thus can add another artifact to the impedance measurements. While a needle array presents its own challenges (e.g., some inevitable muscle trauma), this added variable can be avoided by reducing the number of consecutive impedance measurements per animal. In addition, we designed our array using a modified set of 4 subdermal needles, each coated with nail polish, leaving only approximately 1mm of the needle tip exposed. Using such coated subdermal needles is preferable to standard Teflon-coated monopolar needles, since in our experience the latter tend to produce greater muscle injury at the junction of the needle tip and Teflon insulation.

This study represents the first use of intra-muscular EIM to assess muscle inflammation; however, several limitations remain. First, we followed animals only up to 72 hours post injection and did not detect macrophages in the tissue at this time; therefore, our findings are only related to the initial acute phase of inflammation. Second, our characterization of the inflammatory response was solely based on the quantification of basophilic staining in the muscle(no specific staining was performed to identify neutrophils), and further experimentation could investigate the possibility of correlating EIM parameters to neutrophil content or serological markers to increase the accuracy of this method. Third, the λ-carrageenan model of inflammation is decidedly simple and represents only a single episode of toxic inflammation and none of the longer-term sequelae that would be anticipated with repeated exposure or in other conditions (for example, autoimmune myositis), including scarring and denervation. Fourth, although our histological analyses were blinded to both treatment and duration, the EIM data collection itself was not double blinded. Indeed, while investigators had to inject either the treatment or the vehicle, steps were taken to ensure that terminal EIM measurements were collected without knowledge of the treatment received. It is also important to recognize that the actual data, as displayed on the EIM device, is challenging to interpret until it is downloaded and after complete analysis. Finally, when compared to PBS-injected controls that received the same volume, we were not able to detect the immediate effects of λ-carrageenan (up to 24 hours) and noticed a similar response in all groups. However, such an early response to an increase in tissue volume is anticipated based on past literature [10].

Nevertheless, this work represents a first step towards a better understanding of the relationship between inflammatory changes in muscle and alterations in electrical impedance values. Planned future work will focus on assessing a more physiological model of myositis—namely a model of autoimmune myositis and studying EIM’s ability to detect the impact of therapy.
